# Supplementary material for: Synthesis of Arylene Ether-Type Hyperbranched Poly(triphenylamine) for Lithium Battery Cathodes
Source: Materials (Basel). 2021 Dec 20;14(24):7885. doi: 10.3390/ma14247885 (PMC8707362; doi:10.3390/ma14247885)
Supplement: Supplementary file 1 [file materials-14-07885-s001.zip › materials-1464327-supplementary.pdf]

Supporting Information

Synthesis of Arylene Ether-Type Hyperbranched Poly (Triphenylamine) for Lithium Battery Cathodes

Inah Kang [1,†], Taewoong Lee [2,†], Young Rok Yoon [1], Jee Woo Kim [3], Byung-Kwon Kim [3], Jinhee Lee [1,4,*], Jin Hong Lee [2,*], Sang Youl Kim [1,*]

[1] *Department of Chemistry, Korea Advanced Institute of Science and Technology (KAIST), Daejeon 34141, Korea*

[2] *School of Chemical Engineering, Pusan National University, Busan 46421, Korea*

[3] *Department of Chemistry and Nanoscience, Ewha Womans University, 52, Seoul, 03760, Korea*

[4] *Reliability Assessment Center for Chemical Materials, Korea Research Institute of Chemical Technology (KRICT), Daejeon 34114, Korea*

[†] Inah Kang and Taewoong Lee contributed equally to this work.

a

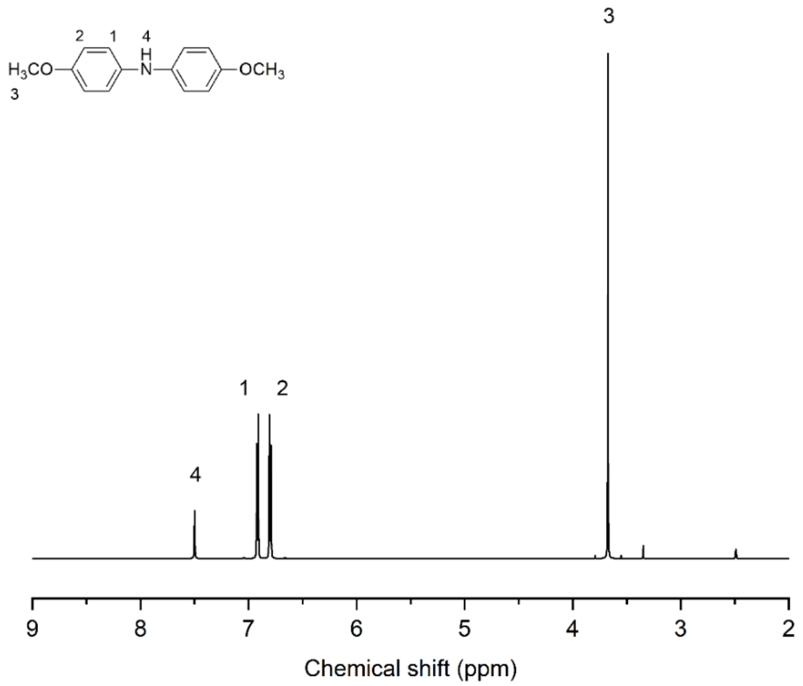

b

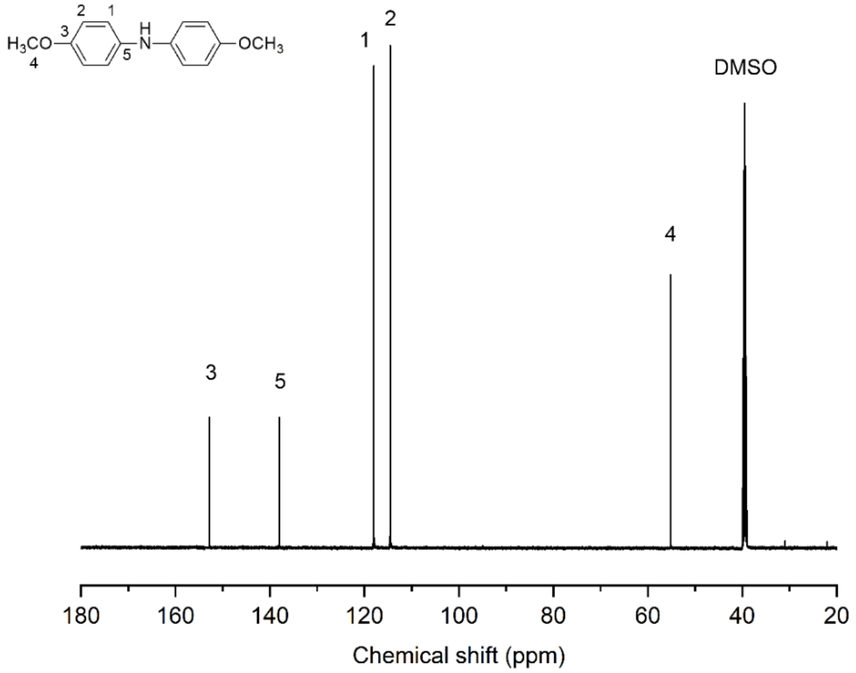

Figure S1. (a) ^{1}H NMR spectra of 1 (DMSO-*d*6, 600 MHz). (b) ^{13}C NMR spectra of 1 (DMSO-*d*6, 150 MHz).

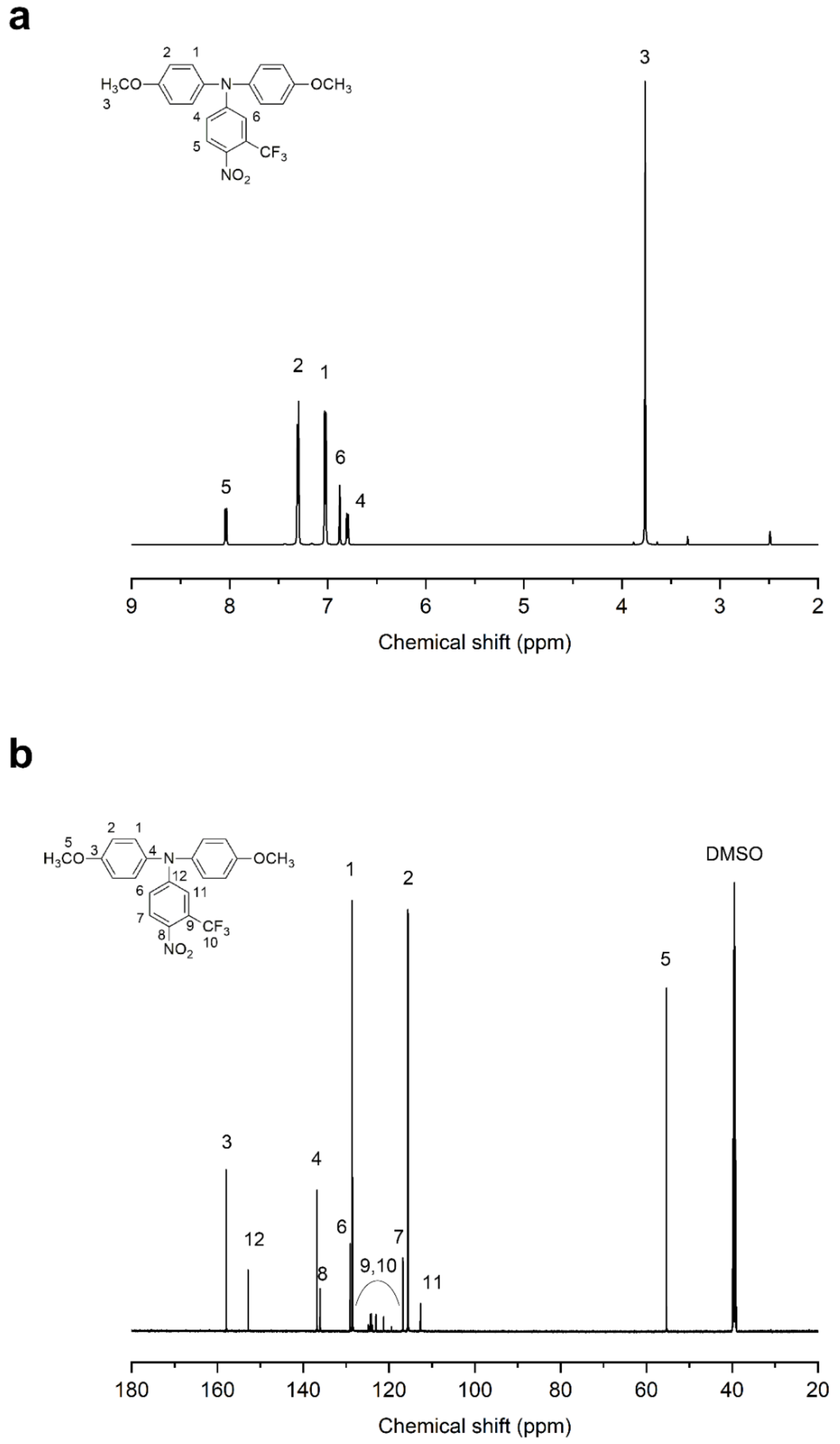

Figure S2. (a) ^{1}H NMR spectra of 2 (DMSO-*d*6, 600 MHz). (b) ^{13}C NMR spectra of 2 (DMSO-*d*6, 150 MHz).

a

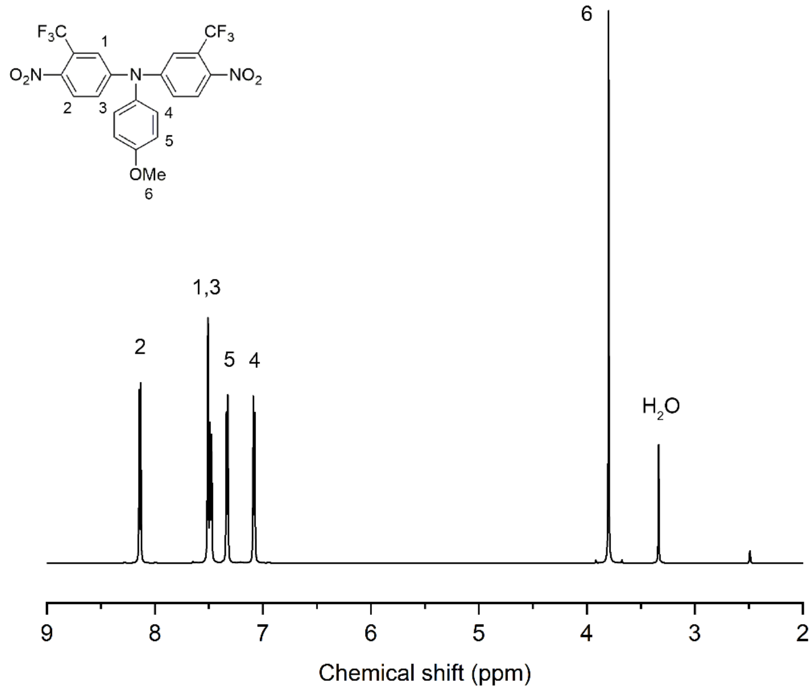

b

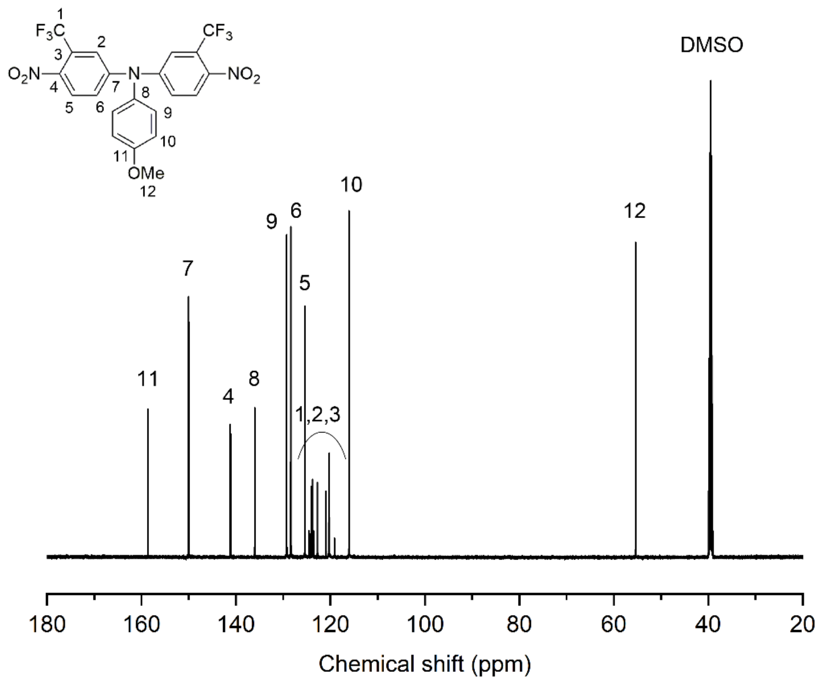

Figure S3. (a) ^{1}H NMR spectra of 3 (DMSO-*d*6, 600 MHz). (b) ^{13}C NMR spectra of 3 (DMSO-*d*6, 150 MHz).

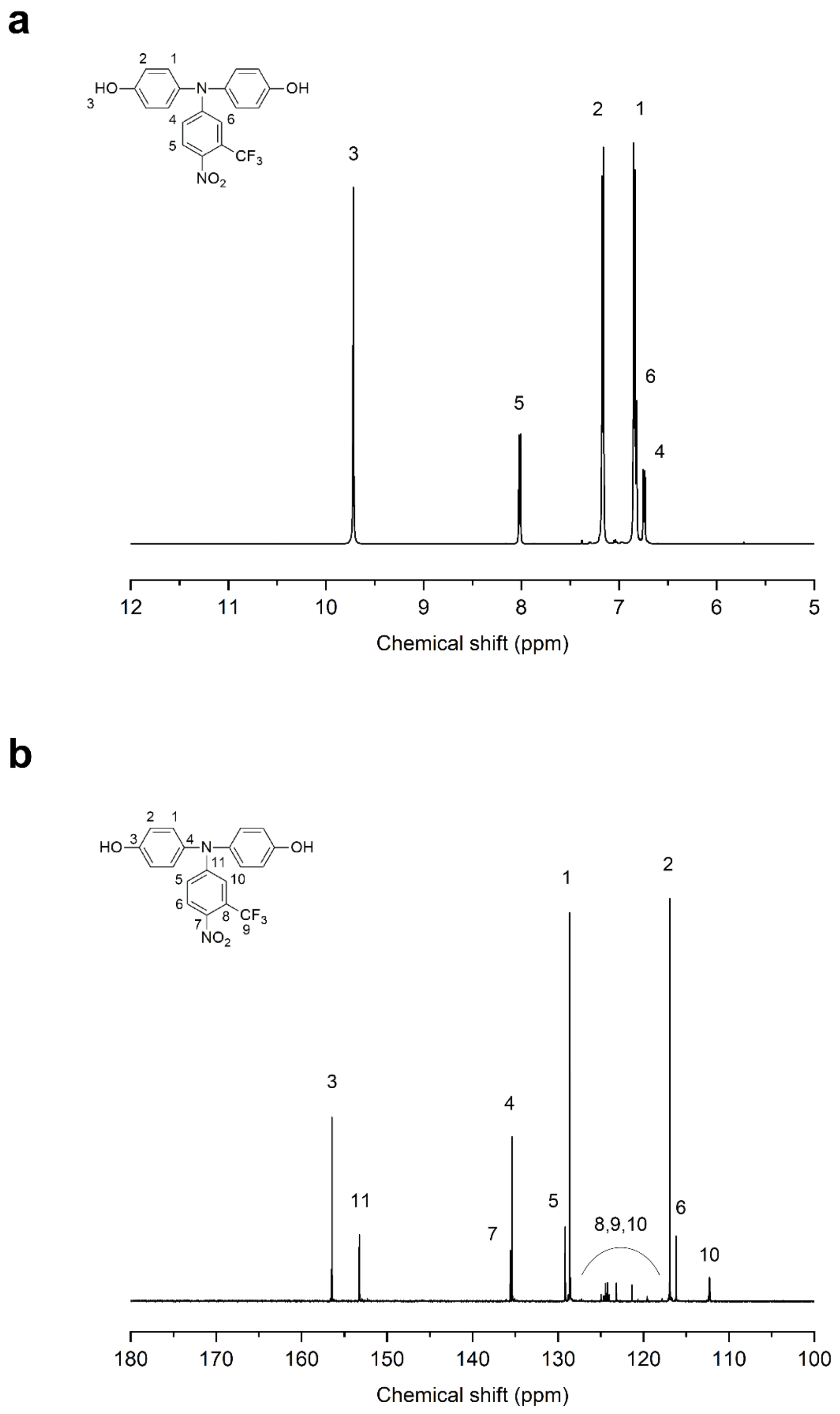

Figure S4. (a) ^{1}H NMR spectra of 4 (DMSO-*d*6, 600 MHz). (b) ^{13}C NMR spectra of 4 (DMSO-*d*6, 150 MHz).

a

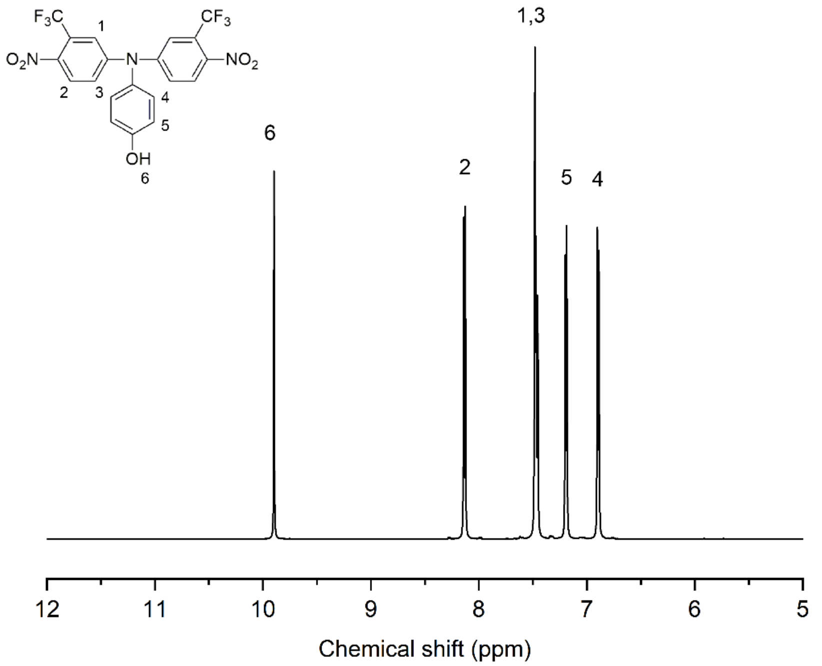

b

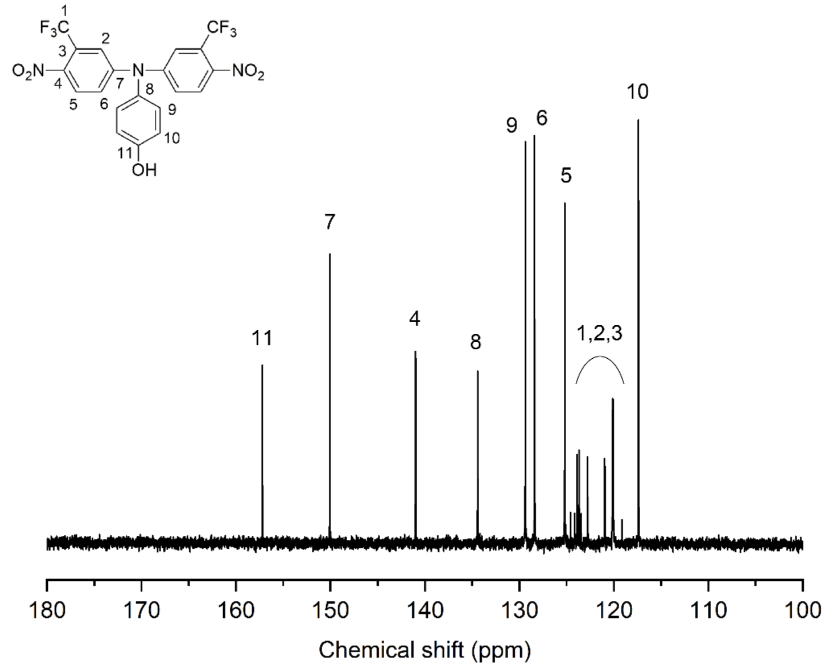

Figure S5. (a) ^{1}H NMR spectra of 5 (DMSO-*d*6, 600 MHz). (b) ^{13}C NMR spectra of 5 (DMSO-*d*6, 150 MHz).

a

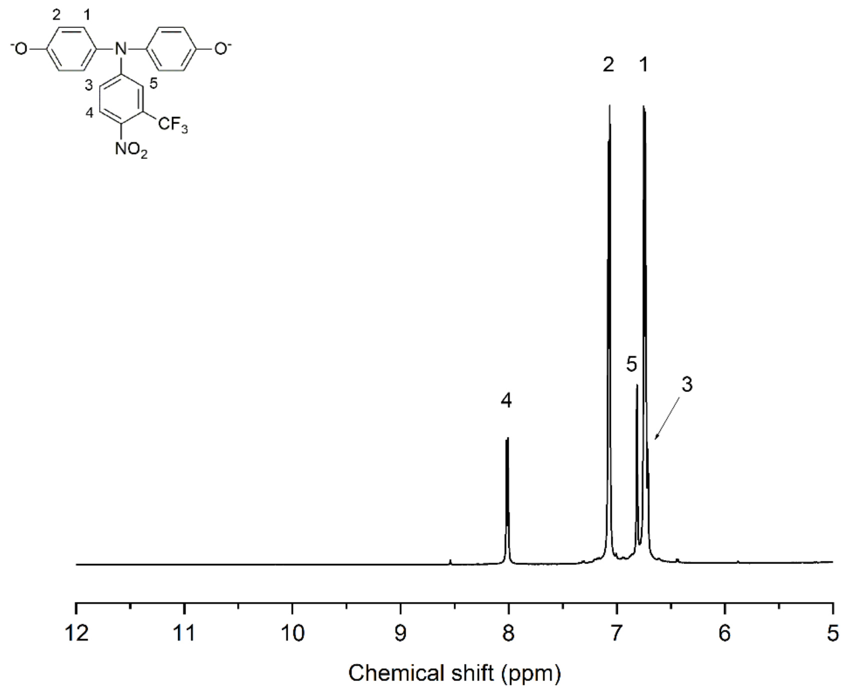

b

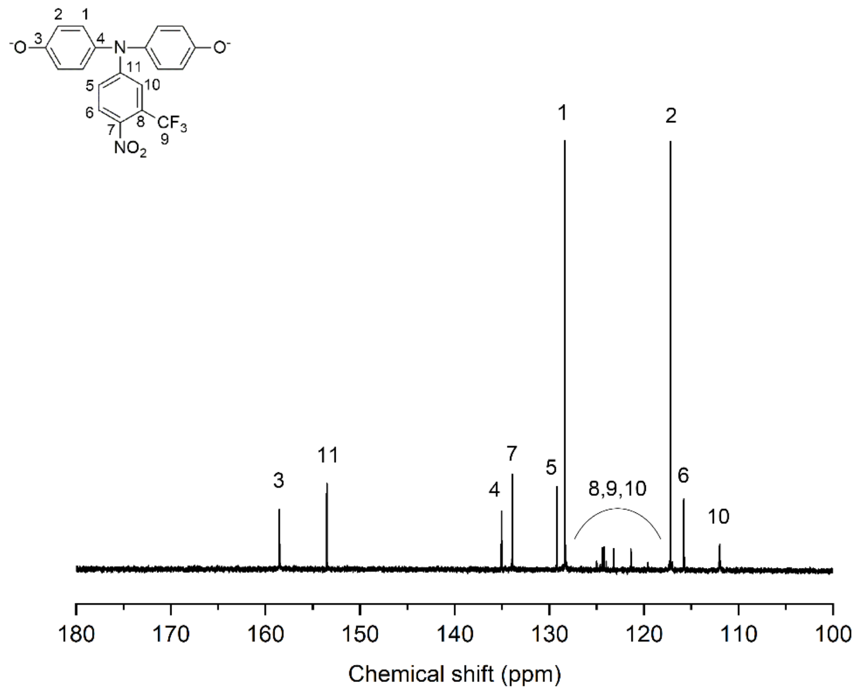

Figure S6. (a) ^{1}H NMR spectra of 4-K (DMSO-*d*6, 600 MHz). (b) ^{13}C NMR spectra of 4-K (DMSO-*d*6, 150 MHz).

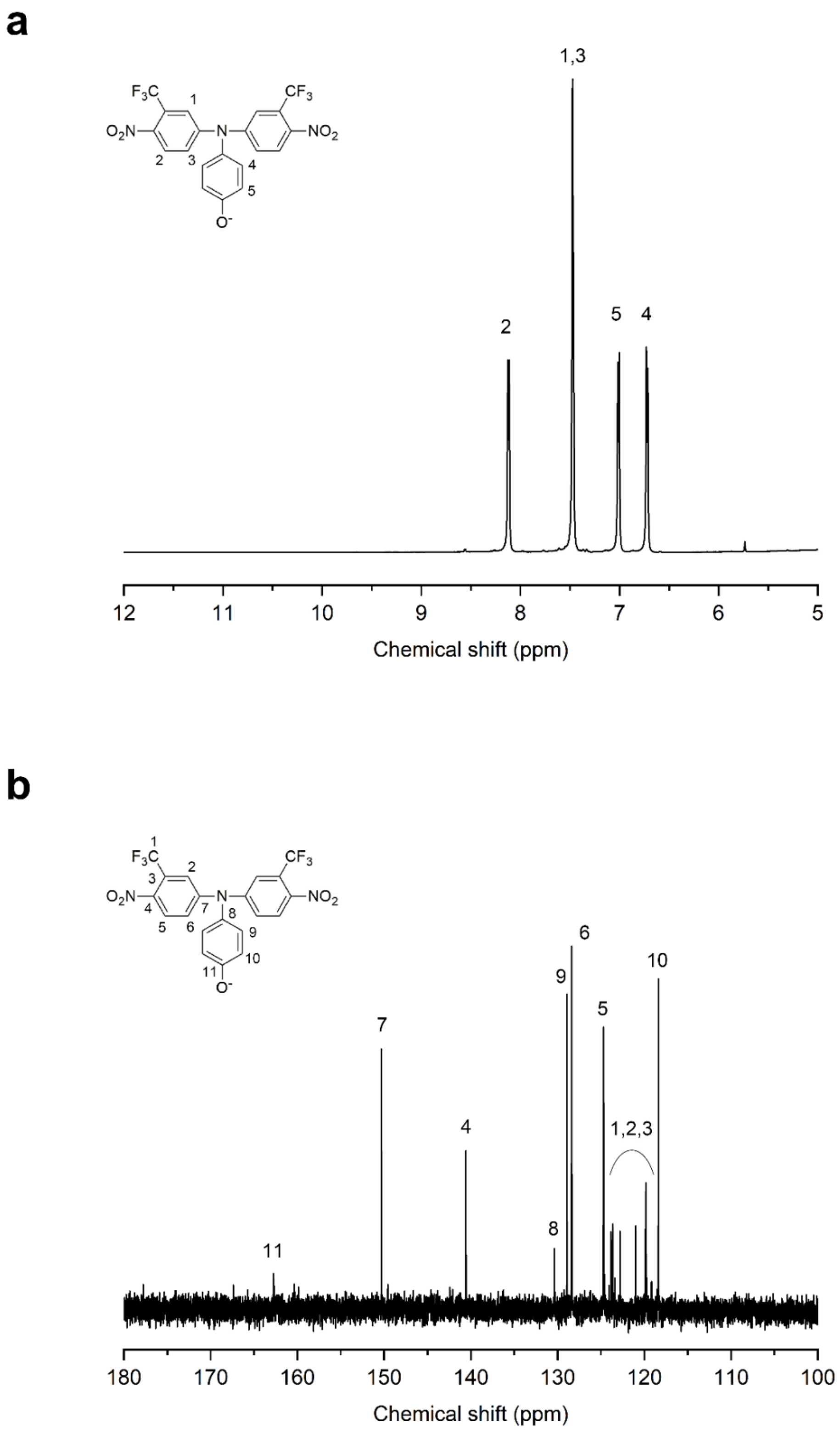

Figure S7. (a) ^{1}H NMR spectra of 5-K (DMSO-*d*6, 600 MHz). (b) ^{13}C NMR spectra of 5-K (DMSO-*d*6, 150 MHz).

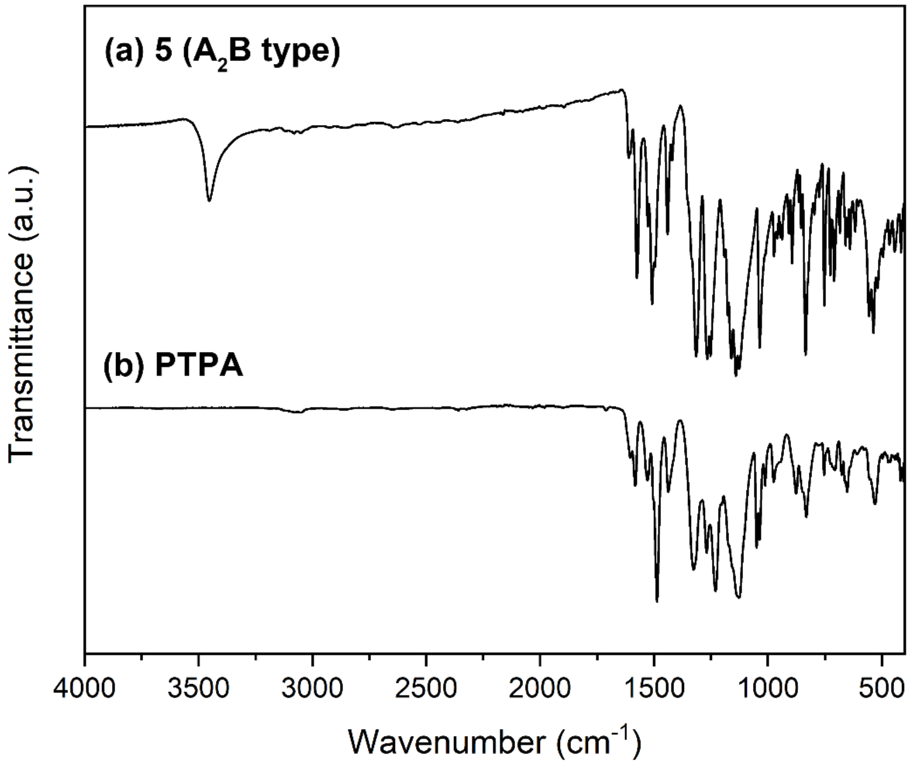

Figure S8. IR spectra of (a) 5 and (b) PTPA.

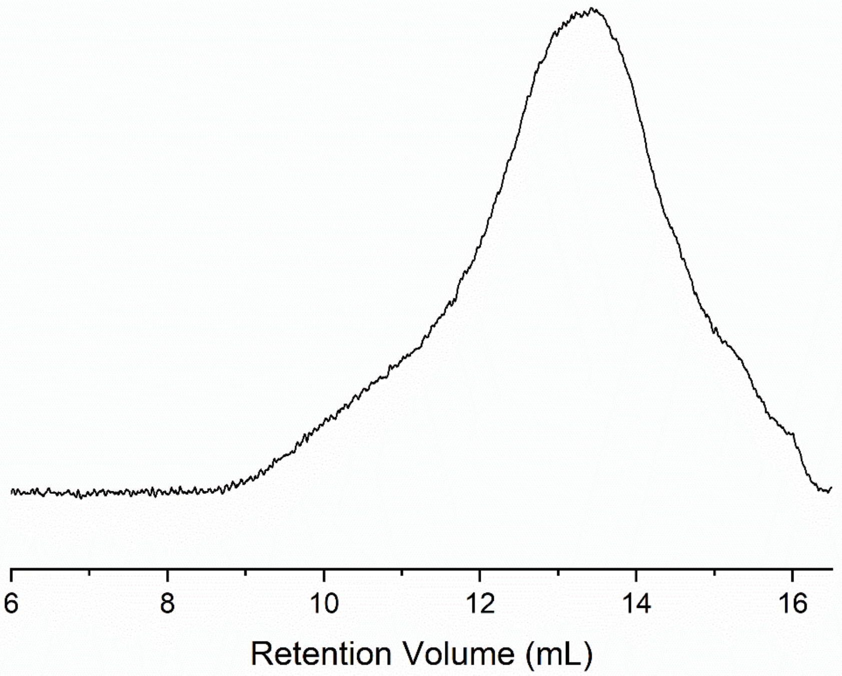

Figure S9. SEC traces of PTPA (THF, 36 °C, 0.6 mL/min). Estimated molecular weight was M_n = 22,200 and M_w = 303,400.

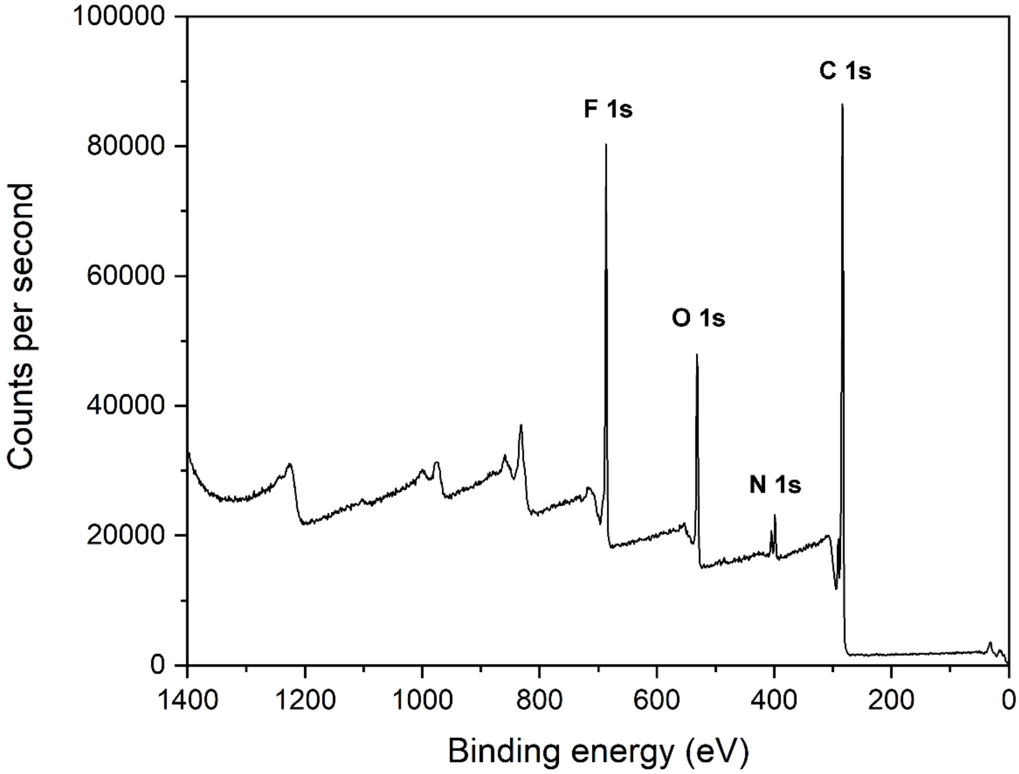

Figure S10. XPS spectrum of PTPA.

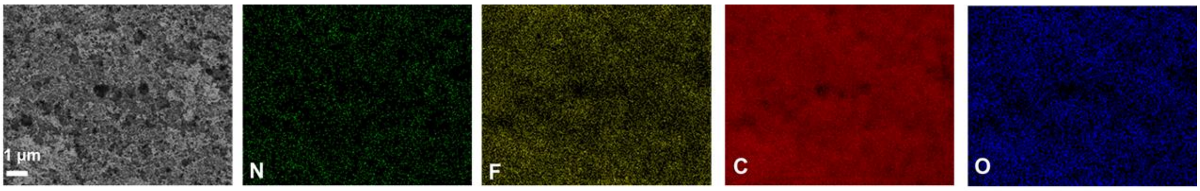

Figure S11. SEM images and element mapping images of PTPA cathode.
